# Supplementary material for: Optimal fluorescent protein tags for quantifying protein oligomerization in living cells
Source: Sci Rep. 2018 Jul 13;8:10634. doi: 10.1038/s41598-018-28858-0 (PMC6045628; doi:10.1038/s41598-018-28858-0)
Supplement: Supplementary file 1 — Supplementary Information [file 41598_2018_28858_MOESM1_ESM.pdf]

Supplementary Material to:

Optimal fluorescent protein tags for quantifying protein oligomerization in living cells

Valentin Dunsing[+1], Madlen Luckner[+2], Boris Zühlke[1], Roberto A. Petazzi[1], Andreas Herrmann[2] and Salvatore Chiantia*[1]

[1] Institute for Biochemistry and Biology, Universität Potsdam, Karl-Liebknecht-Str. 24-25, 14476 Potsdam
[2] Institute for Biology, IRI Life Sciences, Humboldt-Universität zu Berlin, Invalidenstraße 42, 10115 Berlin

[+] These authors contributed equally
* Corresponding author

Brightness analysis in different cell types and at different temperatures

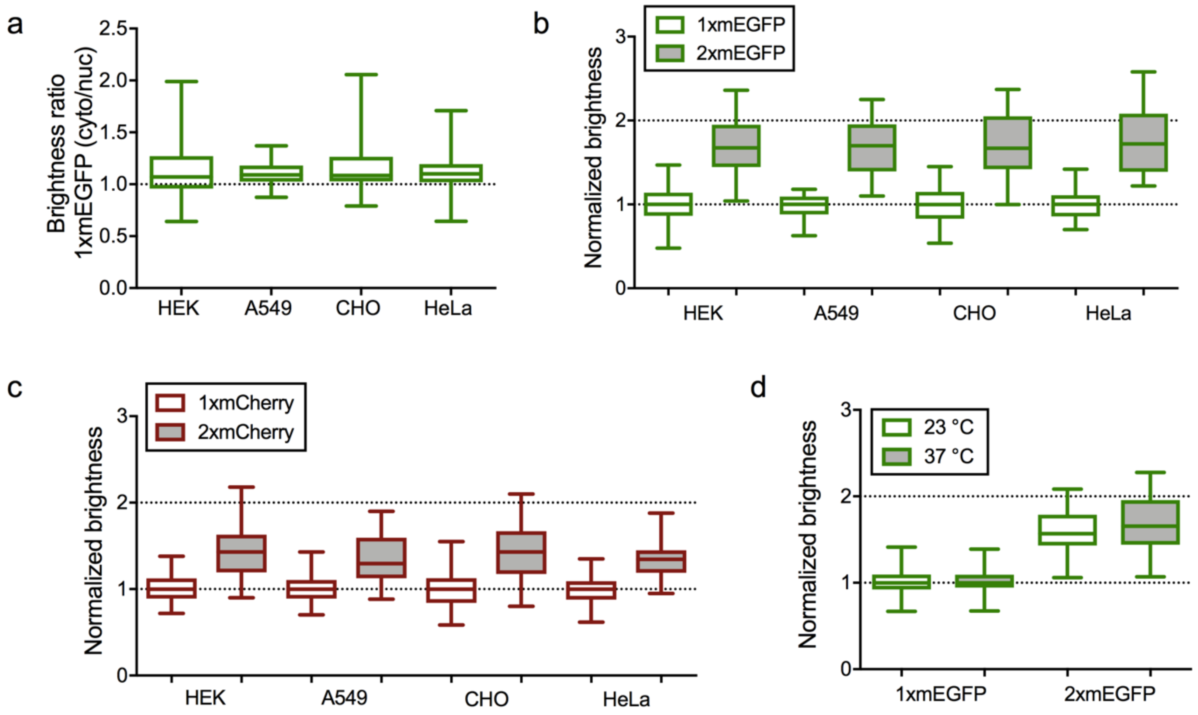

Supplementary Figure S1. Brightness analysis in different cell types and at different temperatures. a: Box plots of brightness ratio of mEGFP in cytoplasm and nucleus of HEK 293T, A549, CHO and HeLa cells, measured with N&B. The ratio was calculated for each single cell measurement. Data represent results of at least three independent experiments (HEK: n=47 cells, A549: n=37 cells, CHO: n=34 cells, HeLa: n=38 cells). **b:** Box plots of normalized molecular brightness of mEGFP monomers (white boxes) and homo-dimers (grey boxes) measured with N&B in cytoplasm of HEK 293T, A549, CHO and HeLa cells. Data represent results of at least three independent experiments (1x HEK: n=47 cells, 2x HEK: n=48 cells, 1x A549: n=38 cells, 2x A549: n=33 cells , 1x CHO: n=36 cells, 2x CHO: n=39 cells , 1x HeLa: n=39 cells, 2x HeLa: n=37 cells). **c:** Box plots of normalized molecular brightness of mCherry monomers and homo-dimers measured with N&B in cytoplasm of HEK 293T, A549, CHO and HeLa cells. Data represent results of at least three independent experiments (1x HEK: n=50 cells, 2x HEK: n=53 cells, 1x A549: n=37 cells, 2x A549: n=36 cells, 1x CHO: n=44 cells, 2x CHO: n=41 cells, 1x HeLa: n=35 cells, 2x HeLa: n=36 cells). **d:** Box plots of normalized molecular brightness of mEGFP monomers and homo-dimers at 23°C (white boxes) and 37°C (grey boxes) measured with pFCS in HEK 293T. Data are pooled from at least three independent experiments (1x 23°C: n=39 cells, 2x 23°C: n=38 cells , 1x 37°C: n=64 cells, 2x 37°C n=63 cells).

Diffusion and FLIM analysis of mEGFP homo-oligomers

In the FCS analysis of the mEGFP homo-oligomer diffusion behavior in cells, introduction of an anomaly parameter α was needed to appropriately fit a 3-D diffusion model (Supplementary Figure S2a). As previously shown[1], we reproducibly observed anomalous subdiffusion in the molecular crowed cytoplasm of cells, indicated by α values lower than one for all investigated mEGFP homo-oligomers (Supplementary Figure S2c). Interestingly, we observed the same average α values for all homo-oligomers. The diffusion times increased for increasing homo-oligomer size (1xmEGFP: 27 kDa, 2xmEGFP: 54 kDa, 3xmEGFP: 81 kDa, 4xmEGFP: 108 kDa) (Supplementary Figure S2c), although with a different scaling than observed *in vitro*[2], in accordance with previous studies[3].

Additionally, we performed fluorescence lifetime imaging measurements (FLIM) in A549 cells, since energy transfer between different mEGFP brightness states or to non-fluorescent subunits could result in a reduction of homo-oligomer brightness values. All lifetime decays could be fitted by a single exponential component (Supplementary Figure S2e), indicating a single mEGFP brightness state in all mEGFP homo-oligomers. We observed negligible lifetime differences for all the tested homo-oligomers (Supplementary Figure S2e) corresponding to FRET efficiencies of less than 1.5%. Thus, we can exclude energy transfer to non-fluorescent mEGFP states.

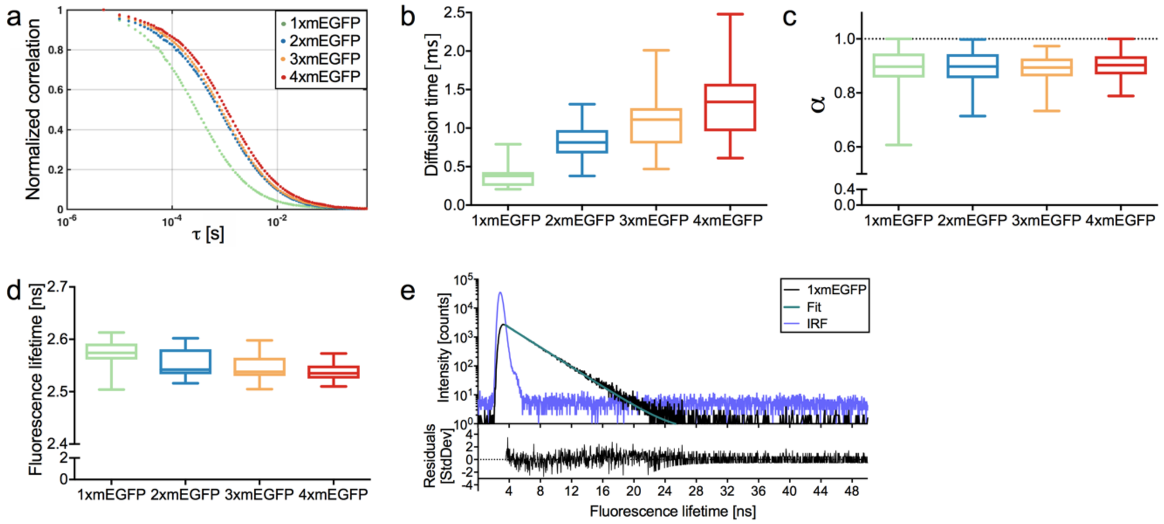

Supplementary Figure S2. Diffusion and FLIM analysis of mEGFP homo-oligomers. a: Normalized average autocorrelation functions (ACFs) of 1xmEGFP, 2xmEGFP, 3xmEGFP and 4xmEGFP (i.e. monomers to tetramers), obtained by pFCS measurements in the cytoplasm of A549 cells. Average ACFs of all cells, measured in at least three independent experiments (1xmEGFP: n=52 cells, 2xmEGFP: n=39 cells, 3xmEGFP: n=42 cells, 4xmEGFP: n=28 cells) are shown. **b:** Box plots of diffusion times of mEGFP homo-oligomers obtained from fitting a model for three-dimensional anomalous diffusion to the data, pooled from all measured cells. **c**: Box plots of anomaly parameter α obtained from the anomalous diffusion model, pooled from all measured cells. **d:** Box plots of fluorescence lifetime of 1xmEGFP (n=16 cells), 2xmEGFP, (n=20 cells), 3xmEGFP (n=15 cells), 4xmEGFP (n=8 cells) measured in two independent experiments in the cytoplasm of A549 cells. **e:** Representative lifetime histogram for 1xmEGFP fitted with a single exponential component fit. The instrument response function (IRF) was calibrated as described in the methods paragraph.

Diffusion analysis of Influenza A virus hemagglutinin (HA) measured with sFCS

From the sFCS auto-correlation functions (Supplementary Figure S3a-d) we also determined the diffusion dynamics of HA-wt-mEGFP (HA-wt) and HA-TMD-mEGFP (HA-TMD). In line with the Saffman-Delbrück model[4], albeit having a different oligomeric state, both constructs display only slight differences in their diffusion coefficients: D_{HA}=0.38 ± 0.01 µm^2/s and $D_{HA\text{-}TMD}$=0.42 ± 0.02 µm^2/s (Supplementary Figure S3e).

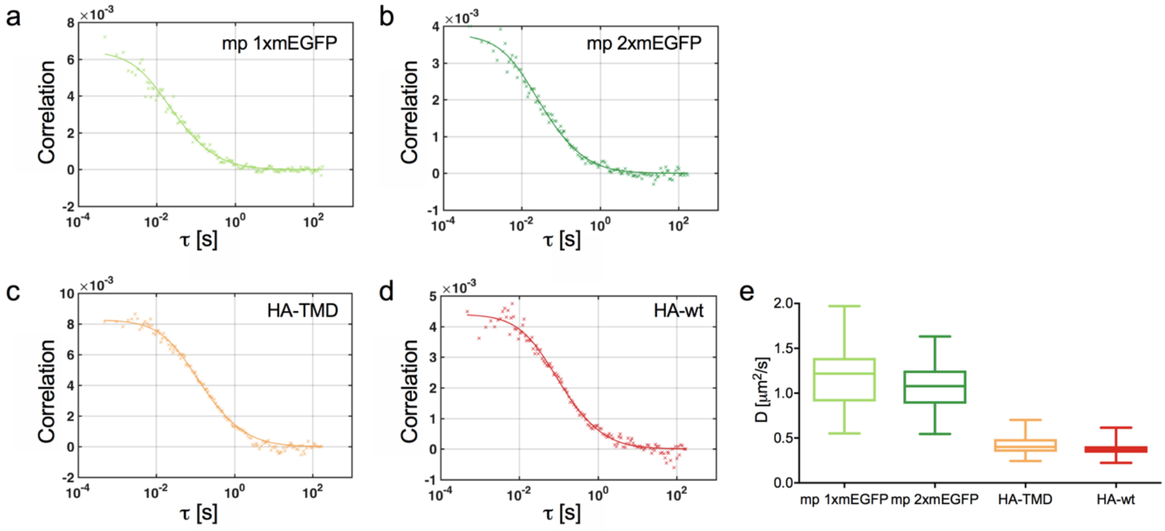

Supplementary Figure S3. Diffusion analysis of Influenza A virus hemagglutinin (HA) measured with sFCS. a-d: Representative correlation functions and fit curves for sFCS measurements of mp 1xmEGFP (a), mp 2xmEGFP (b), HA-TMD-mEGFP (c) and HA-wt-mEGFP (d) measured in HEK 293T cells. Fit curves (solid lines) were obtained by fitting a two-dimensional diffusion model to the data. **e:** Box plots of diffusion coefficients calculated from sFCS diffusion times, pooled from at least three independent experiments (mp 1xmEGFP: n=55 cells, mp 2xmEGFP: n=54 cells, HA-TMD: n=37 cells, HA-wt: n=36 cells).

Comparison of mCherry2 to mCherry brightness values in different cell types and at different temperatures

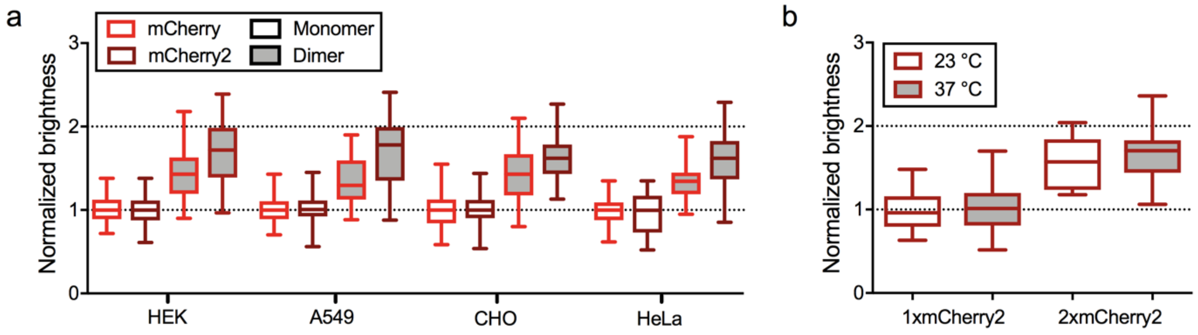

Supplementary Figure S4. Comparison of mCherry2 to mCherry brightness values in different cell types and at different temperatures. a: Box plots of normalized molecular brightness of mCherry (light red)/ mCherry2 (dark red) monomers (white boxes) and homo-dimers (grey boxes) measured with N&B in the cytoplasm of HEK 293T, A549, CHO and HeLa cells. Data represent results of at least three independent experiments (mCherry: 1x HEK: n=50 cells, 2x HEK: n=53 cells, 1x A549: n=37 cells, 2x A549: n=36 cells , 1x CHO: n=44 cells, 2x CHO: n=41 cells , 1x HeLa: n=35 cells, 2x HeLa: n=36 cells; mCherry2: 1x HEK: n=49 cells, 2x HEK: n=54 cells, 1x A549: n=38 cells, 2x A549: n=34 cells , 1x CHO: n=43 cells, 2x CHO: n=40 cells , 1x HeLa: n=38 cells, 2x HeLa: n=40 cells). **b**: Box plots of normalized molecular brightness of mCherry2 monomers and homo-dimers at 23°C (white boxes) and 37°C (grey boxes) measured with pFCS in HEK 293T. Data are pooled from at least two independent experiments (1x 23°C: n=18 cells , 2x 23°C: n=20 cells , 1x 37°C: n=34 cells, 2x 37°C n=33 cells).

Relative cross-correlation of mCherry-/mCherry2-mEGFP hetero-dimers and negative controls

Based on the determined apparent fluorescence probabilities ($p_{f,mEGFP}$=0.65, $p_{f,mCherry}$=0.42, $p_{f,mCherry2}$=0.64), the expected relative populations of fluorescent and non-fluorescent mCherry-/mCherry2-mEGFP hetero-dimers can be calculated. For mCherry-mEGFP, the following relative populations f are expected in the sample: $f_{G-R}= p_{f,mEGFP} \cdot p_{f,mCherry}$=27%, f_{g-R}=15%,

f_{G-r}=38%, f_{g-r}=20%, where G, R denote fluorescent and g, r non-fluorescent mEGFP/mCherry FPs. Thus, the expected relative concentration of fluorescent mCherry hetero-dimer subunits (relative to the concentration of those containg flurescent mEGFP) is $\frac{f_{G-R}+f_{g-R}}{f_{G-R}+f_{G-r}} = 0.65$, which agrees very well with the measured average ratio of the mEGFP and mCherry ACF amplitudes of 0.65 (Figure 5). For mCherry2-mEGFP hetero-dimers, the expected populations are:

$f_{G-R}=p_{f,mEGFP} \cdot p_{f,mCherry2}$=42%, f_{g-R}=22%, f_{G-r}=23%, f_{g-r}=12%.

Thus, the relative concentration of fluorescent mEGFP and mCherry2 is expected to be $\frac{f_{G-R}+f_{g-R}}{f_{G-R}+f_{G-r}} = 0.98$, which agrees well with the measured value of 0.97 (Figure 5). Based on the p_f values and corresponding populations, it is also expected that the relative cross-correlation (rel.cc.) of mCherry-/ and mCherry2-mEGFP hetero-dimers is similar, since this quantity depends on the relative amount of FPs in a complex, for the less abundant FP species:

$$\frac{rel.cc._{mCherry2-mEGFP}}{rel.cc._{mCherry-mEGFP}} = \frac{max\left\{\frac{f_{G-R}}{f_{G-R}+f_{G-r}}, \frac{f_{G-R}}{f_{G-R}+f_{g-R}}\right\}_{mCherry2}}{max\left\{\frac{f_{G-R}}{f_{G-R}+f_{G-r}}, \frac{f_{G-R}}{f_{G-R}+f_{g-R}}\right\}_{mCherry}} = 1.02$$

In agreement with this expected ratio, mCherry-/ mCherry2-mEGFP hetero-dimers show the same relative cross-correlation in pFCCS measurements of A549 cells: $rel.cc._{mCherry-mEGFP}=0.39 \pm 0.02$, $rel.cc._{mCherry2-mEGFP}=0.39 \pm 0.01$. As a negative control, pFCCS

measurements were performed in A549 cells co-expressing mCherry (or mCherry2) and mEGFP. The obtained relative cross-correlation is close to zero ($rel.cc._{mCherry+mEGFP}=0.06 \pm 0.01$, $rel.cc._{mCherry2+mEGFP}=0.04 \pm 0.01$). The residual positive cross-correlation is most likely due to minor cross-talk of mEGFP emission into the mCherry/ mCherry2 channel.

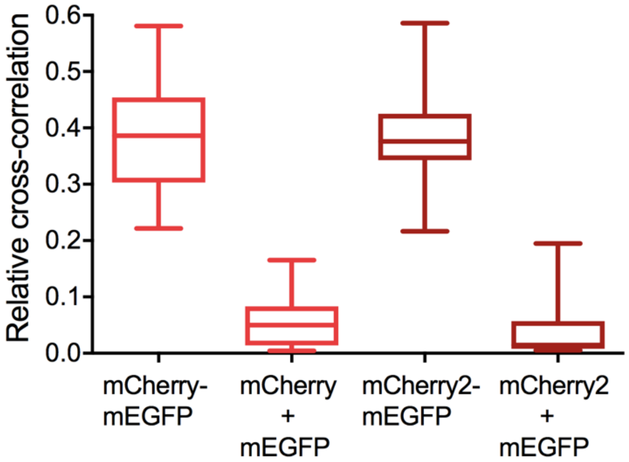

Supplementary Figure S5. Relative cross-correlation of mCherry-/mCherry2-mEGFP hetero-dimers and negative controls. Box plots of cross-correlation values measured for mCherry-mEGFP (light red) and mCherry2-mEGFP (dark red) hetero-dimers measured in A549 cells. Negative controls for cross-correlation were obtained by co-expressing mCherry (or mCherry2) together with mEGFP in A549 cells. The relative cross-correlation was calculated for each measurement as described in Methods. Data are pooled from at least two independent experiments (mCherry-mEGFP: n=35 cells, mCherry+mEGFP: n=19 cells, mCherry2-mEGFP: n=32 cells, mCherry2+mEGFP: n=16 cells)

Supplementary Table S1: Apparent fluorescence probabilities (p_f) determined in this study. Values are given as mean ± SEM, NA: values were not determined.

Cell type	Localization	Method	mEGFP	mEYFP	mCherry	mCherry2	mCardinal	mRuby3	mScarlet	mScarlet-I
**A549**	Cytoplasm	N&B	0.68 ± 0.06	NA	0.36 ± 0.05	0.69 ± 0.07	NA	NA	NA	NA
		pFCS	0.65 ± 0.05	0.47 ± 0.07	0.42 ± 0.07	0.64 ± 0.09	NA	NA	NA	NA
**CHO**	Cytoplasm	N&B	0.72 ± 0.06	NA	0.44 ± 0.05	0.62 ± 0.04	NA	NA	NA	NA
**HEK 293T**	Cytoplasm	N&B	0.69 ± 0.05	0.63 ± 0.05	0.41 ±0.04	0.71 ± 0.05	0.24 ± 0.03	0.22 ± 0.05	0.4 ± 0.05	0.63 ± 0.05
		pFCS	0.84 ± 0.06	0.62 ± 0.04	0.45 ± 0.06	NA	NA	NA	NA	NA
	Nucleus	N&B	0.76 ± 0.06	0.59 ± 0.07	0.3 ± 0.03	NA	NA	NA	NA	NA
	Plasma membrane	sFCS	0.67 ± 0.04	0.44 ± 0.04	0.43 ± 0.05	NA	NA	NA	NA	NA
**HeLa**	Cytoplasm	N&B	0.76 ± 0.06	NA	0.34 ± 0.03	0.63 ± 0.05	NA	NA	NA	NA
**U2OS**	Cytoplasm	N&B	0.72 ± 0.05	NA	NA	NA	NA	NA	NA	NA

Supplementary Methods

Fluorescent protein constructs. For the cloning of all following constructs, standard PCRs with custom-designed primers (Supplementary Table S2) were performed to obtain monomeric FP cassettes, followed by digestion with fast digest restriction enzymes and ligation with T4-DNA-Ligase according to the manufacturer's instructions. All enzymes were purchased from Thermo Fisher Scientific, unless specified otherwise.

The constructs 2xmEGFP, 3xmEGFP and 4xmEGFP (i.e. mEGFP homo-dimer, homo-trimer and homo-tetramer) were obtained by step-wise cloning of monomeric mEGFP cassettes amplified from mEGFP-N1, a gift from Michael Davidson (Addgene plasmid #54767). First, 2xmEGFP was generated by ligating a mEGFP cassette into mEGFP-N1 digested with BamHI and AgeI. Subsequently, an additional monomeric mEGFP cassette was ligated into 2xmEGFP by digestion with KpnI and BamHI to generate 3xmEGFP. Finally, 4xmEGFP was obtained by ligation of an additional monomeric mEGFP cassette into 3xmEGFP by digestion with EcoRI and KpnI. All mEGFP subunits are linked by a polypeptide sequence of five amino acids. To ensure purity of mEGFP homo-oligomers, all full-length inserts were subcloned into pcDNA^{TM}3.1(+) (Thermo Fisher Scientific) possessing ampicillin instead of kanamycin resistance.

The GlnA-mEGFP plasmid was a kind gift from Ana García Sáez (University of Tübingen) and cloned based on pGlnA-Ypet (gift from Mike Heilemann, Addgene plasmid #98278).

To obtain 2xmEYFP, mEYFP was amplified from mEYFP-N1[5] and inserted into mEYFP-C1 by digestion with KpnI and BamHI.

The plasmids mCherry-C1 and mCherry2-C1/N1 (gifts from Michael Davidson, Addgene plasmids #54563 and #54517, respectively) were used to generate 2xmCherry and 2xmCherry2, respectively. First, mCherry-C1 was generated by amplification of mCherry from mCherry-pLEXY plasmid (a gift from Barbara Di Ventura & Roland Eils, Addgene plasmid #72656) and inserted into a pBR322 empty vector. A second mCherry cassette was

inserted into this vector by digestion with XhoI and BamHI to obtain 2xmCherry (i.e. mCherry homo-dimer). The 2xmCherry2 (mCherry2 homo-dimer) plasmid was generated by amplification of mCherry2 and insertion of this construct into mCherry2-C1 through digestion with XhoI and BamHI. To clone mRuby3-C1, mRuby3 was amplified from the pKanCMV-mClover3-mRuby3 plasmid, a gift from Michael Lin (Addgene plasmid #74252). The obtained PCR product was digested with AgeI and XhoI and exchanged with mEYFP from digested mEYFP-C1 plasmid. For 2xmRuby3 (mRuby3 homo-dimer), mRuby3 was again amplified by PCR and the product inserted into mRuby3-C1 by digestion with KpnI and BamHI. The mCardinal-C1/N1 plasmids were a gift from Michael Davidson (Addgene plasmids #54590 and #54799). To obtain 2xmCardinal (mCardinal homo-dimer), mCardinal was amplified from mCardinal-C1 and the PCR product inserted into mCardinal-C1 by digestion with KpnI and BamHI. The plasmids mScarlet-C1 and mScarlet-I-C1 are gifts from Dorus Gardella (Addgene plasmids #85042 and #85044). 2xmScarlet (mScarlet homo-dimer) and 2xmScarlet-I (mScarlet-I homo-dimer) were generated by amplification of mScarlet and mScarlet-I from the corresponding plasmids and reintegration into mScarlet-C1 and mScarlet-I-C1 by digestion with XhoI and KpnI. To ensure purity of homo-dimers, all full-length homo-dimers were subcloned into pcDNA^{TM}3.1(+) (Thermo Fisher Scientific) possessing ampicillin instead of kanamycin resistance.

The hetero-dimers mCherry-mEGFP and mCherry2-mEGFP were generated by amplification of mCherry and mCherry2, respectively, and insertion of the obtained constructs into mEGFP-C1, (Michael Davidson, Addgene plasmid #54759), by digestion with XhoI and BamHI. Both fluorophores are linked by five and seven amino acids, respectively.

The membrane constructs consisting of mEGFP linked to a myristoylated and palmitoylated peptide (mp 1xmEGFP) and its homo-dimer mp 2xmEGFP were kind gifts from Richard J. Ward (University of Glasgow)[6]. The analogue mp 1xmEYFP construct was obtained as described elsewhere[5]. To generate mp 2xmEYFP, the 2xmEYFP cassette described above was

transferred into a mp mCardinal vector[7], by digestion with AgeI and BamHI. The GPI mCherry (glycosylphosphatidylinositol-anchored mCherry) plasmid was a kind gift from Roland Schwarzer (Gladstone Institute, San Francisco). Based on this plasmid, GPI 2xmCherry was generated by amplification of a mCherry cassette and ligation of the obtained insert into GPI mCherry, digested using SalI and BamHI.

The Influenza virus A/chicken/FPV/Rostock/1934 hemagglutinin (HA) constructs HA-wt-mEGFP and HA-TMD-mEGFP were cloned based on the previously described HA-wt-mEYFP[5] and HA-TMD-mEYFP[8] plasmids. HA-wt-mEYFP contains full-length HA protein fused to mEYFP at the C-terminus, whereas in HA-TMD-mEYFP a large part of the extracellular domain of HA is replaced by mEYFP. To clone HA-wt-mEGFP, HA-wt-mEYFP was digested using BglII and SacII (New England Biolabs) and the obtained HA insert ligated into mEGFP-N1. For HA-TMD-mEGFP, HA-TMD-mEYFP plasmid and mEGFP-N1 vector were digested with AgeI and BsrGI to replace mEYFP with mEGFP.

Supplementary Table S2: Primer sequences (5'-3' orientation) used for cloning of FP expression plasmids.

Construct	Orientation (restriction enzyme)	Sequence (5'-3')
2xmEGFP	forward (BamHI)	GAGAGGGATCCATGGTGAGCAAGGGCGAGGAG
	reverse (AgeI)	GAGAACCGGTCTCTTGTACAGCTCGTCCATGCC
3xmEGFP	forward (KpnI)	GAGAGGTACCATGGTGAGCAAGGGCGAGGAG
	reverse (BamHI)	GAGAGGATCCCGACGGTCTCTTGTACAGCTCGTCCATGCC
4xmEGFP	forward (EcoRI)	GAGAGAATTCATGGTGAGCAAGGGCGAGGAG
	reverse (KpnI)	GAGAGGTACCGACCGGTCTCTTGTACAGCTCGTCCATGCC
mCherry-C1	forward (NheI)	GTACGGCTAGCATGGTGAGCAAGGGCGAGGAG
	reverse (BglII)	GACAGATCTGAGTCCGGACTTGTACAGCTCGTCCATGC
2xmEYFP, 2xmCardinal	forward (KpnI)	ATATGGTACCATGGTGAGCAAGGGC
	reverse (BamHI)	CGCGGGATCCCTTGTACAGCTCGTCC
2xmCherry, 2xmCherry2, mCherry-mEGFP, mCherry2-mEGFP	forward (XhoI)	GAGACTCGAGGAATGGTGAGCAAGGGCGAGGAG
	reverse (BamHI)	GAGAGGATCCTTACTTGTACAGCTCGTCCATGCC
GPI 2xmCherry	forward (SalI)	GAGAGTCGACTATGGTGAGCAAGGGCGAGGAG
	reverse (BamHI)	GAGAGGATCCGAAGCTTGAGCTCGAGATCTAGAAGAACCAGAACCAGTAG ACCAGTACCATGACCTCCGGACTTGTACAGCTCGTCCATGCCGCCGGTG
mRuby3-C1	forward (AgeI)	ATATACCGGTCATGGTGAGCAAAGGC
	reverse (XhoI)	CGCGCTCGAGCTTGTACAGCTCGTCC
2xmRuby3	forward (KpnI)	ATATGGTACCCATGGTGAGCAAAGGC
	reverse (BamHI)	CGCGGGATCCCTTGTACAGCTCGTCC
2xmScarlet(-I)	forward (XhoI)	AGAGTCCTCGAGATATGGTGAGCAAGGGCGAGGCAGTGATC
	reverse (KpnI)	CTGCAGGTACCTTACAGCTCGTCCATGCCGCCGGTG

References

1. Weiss, M., Elsner, M., Kartberg, F. & Nilsson, T. Anomalous subdiffusion is a measure for cytoplasmic crowding in living cells. *Biophys. J.* **87,** 3518–3524 (2004).
2. Vámosi, G. *et al.* EGFP oligomers as natural fluorescence and hydrodynamic standards. *Sci. Rep.* **6,** 33022 (2016).
3. Dross, N. *et al.* Mapping eGFP Oligomer Mobility in Living Cell Nuclei. *PLoS One* **4,** e5041 (2009).
4. Saffman, P. G. & Delbrück, M. Brownian motion in biological membranes. *Proc. Natl. Acad. Sci. U. S. A.* **72,** 3111–3113 (1975).
5. Engel, S. *et al.* FLIM-FRET and FRAP reveal association of influenza virus haemagglutinin with membrane rafts. *Biochem. J.* **425,** 567–573 (2010).
6. Ward, R. J., Pediani, J. D., Godin, A. G. & Milligan, G. Regulation of oligomeric organization of the serotonin 5-hydroxytryptamine 2C (5-HT2C) receptor observed by spatial intensity distribution analysis. *J. Biol. Chem.* **290,** 12844–57 (2015).
7. Dunsing, V., Mayer, M., Liebsch, F., Multhaup, G. & Chiantia, S. Direct evidence of amyloid precursor-like protein 1 trans interactions in cell-cell adhesion platforms investigated via fluorescence fluctuation spectroscopy. *Mol. Biol. Cell* **28,** 3609–3620 (2017).
8. Scolari, S. *et al.* Lateral distribution of the transmembrane domain of influenza virus hemagglutinin revealed by time-resolved fluorescence imaging. *J. Biol. Chem.* **284,** 15708–16 (2009).
